# Influencing factors and enhancement strategies for vaccination behavior among Chinese residents: an empirical analysis based on a nationwide survey

**DOI:** 10.3389/fpubh.2025.1640753

**Published:** 2025-08-20

**Authors:** Meixia Zhang, Xiao Fang, Mingshan Wang, Ning Pan, Hui Wang, Zheng Liu

**Affiliations:** ^1^Department of Urology, Shandong Provincial Hospital Affiliated to Shandong First Medical University, Jinan, China; ^2^School of Nursing and Rehabilitation, Cheeloo Medical College, Shandong University, Jinan, China

**Keywords:** vaccination behavior, influencing factors, cross-sectional survey, multi-factorial analysis, health literacy, personality traits, socioeconomic factors

## Abstract

**Background:**

Vaccination is a cornerstone of healthcare systems, and increasing vaccination coverage is crucial for achieving public health objectives globally. However, vaccine acceptance rates vary considerably across different regions worldwide. In China, understanding the determinants of vaccine acceptance is crucial for enhancing coverage and achieving public health goals.

**Objective:**

This study examined the factors influencing vaccine acceptance among Chinese residents and proposes response strategies to enhance public vaccine acceptance, thereby contributing to public health development.

**Methods:**

Based on cross-sectional data from the 2023 Chinese Residents' Psychological and Behavioral Survey (PBICR), a questionnaire was administered to 30,011 participants using the eHEALS-SF Scale, Health Literacy Scale (HLS-SF4), Big Five Inventory-10 (BFI-10), General Risk Tendency Scale (GRiSP), and Media Motivation Scale. Univariate analysis was performed initially, followed by logistic regression for variables with a *P* ≤ 0.05, to examine the impact of demographic characteristics, health literacy, personality traits, and other factors on vaccination behavior.

**Results:**

Among Chinese residents, 57.23% reported receiving vaccinations. This included 4,555 (15.18%) who received the HPV vaccine, 12,103 (40.33%) the influenza vaccine (IFV), 2,450 (8.16%) the herpes zoster vaccine (HZV), and 9,172 (30.56%) the hepatitis vaccine. Univariate analysis identified 17 factors significantly associated with overall vaccination behavior (*P* < 0.05), including gender, age, education level, disease experience, health literacy, personality traits (e.g., openness and conscientiousness), and media influence. Logistic regression analysis revealed 11 associated factors: being female, aged 45–59 years, having a high education level, being a student, experiencing disease, residing in urban areas, earning high income, possessing high health literacy, exhibiting low openness, showing low risk tendency, and demonstrating high media motivation were positively associated with vaccination behavior (*P* < 0.05). Differences in the influencing factors between vaccines and the interaction of the vaccines are also found in the research.

**Conclusion:**

The acceptance of vaccines among Chinese residents was influenced by a combination of demographic characteristics, health perception, personality traits, and socioeconomic factors. To enhance vaccination coverage, it is essential to implement precise interventions, optimize information dissemination strategies, and provide personalized health services tailored to specific groups, such as those with low educational attainment, individuals residing in rural areas, and those with high openness personality traits.

## 1 Introduction

Vaccines are a cornerstone of infectious disease prevention and are indispensable for safeguarding global public health security (1). This was emphatically demonstrated during the COVID-19 pandemic, where the rapid development and deployment of vaccines proved crucial in curbing viral transmission and reducing severe illness and mortality (2). Nevertheless, the effectiveness of vaccines depends not only on their scientific validity but also on public acceptance. Indeed, vaccine hesitancy—defined by the World Health Organization (WHO) as a delay in acceptance or refusal of vaccination despite availability (3)—poses a significant threat. Recognizing its impact, WHO has listed vaccine hesitancy among the top 10 threats to global health, underscoring how public trust and willingness directly influence the achievement of herd immunity (4). Furthermore, complexities in public decision-making are amplified by factors such as novel vaccine technologies, booster policies, and mixed vaccination regimens (5, 6). To better understand the mechanisms underlying vaccine acceptance, two prominent theoretical frameworks provide valuable insights: the Health Belief Model (HBM) and the Theory of Planned Behavior (TPB) (7, 8). The HBM posits that individual health behavior is driven by five key perceptions: perceived susceptibility to disease, perceived severity of disease consequences, perceived benefits of preventive actions, perceived barriers to behavior, and cues to action. This model helps explain how demographic and experiential factors (e.g., disease history) shape vaccination intentions by influencing risk perception. Complementarily, the TPB emphasizes that behavioral intention is determined by three core factors: attitude toward the behavior (positive or negative evaluations), subjective norms (perceived social pressure to perform the behavior), and perceived behavioral control (perceived ease or difficulty of performing the behavior). TPB provides a framework to analyze how psychological traits (e.g., personality) and social influences (e.g., media) interact to affect vaccination behavior.

Globally, vaccine acceptance exhibits substantial variation across regions and vaccine types. Notably, China stands out as an example of high public trust in vaccines. For instance, a large multi-country study (*n* = 13,426 across 19 nations) published in Nature Medicine reported remarkably high COVID-19 vaccination willingness (87%) and low explicit refusal rates (0.7%) among the Chinese population, ranking highest globally (9). This highlights a generally positive foundation of public cooperation with vaccination efforts in China. Building on this foundation, China's current disease prevention and control strategy prioritizes several key vaccines targeting diseases with significant burden, high social attention, and strong policy support: the human papillomavirus vaccine (HPV), influenza vaccine (IFV), herpes zoster vaccine (HZV), and hepatitis vaccines. Therefore, in this study, “vaccination behavior of Chinese residents” specifically refers to the uptake of these four strategically important vaccines. Examining the landscape of these specific vaccines reveals both progress and persistent challenges:

**HPV Vaccine:** While crucial for preventing cervical cancer (linked to 99.7% of cases), the uptake faces hurdles. Surveys indicate a gap between positive intention (e.g., 40.0% among some student groups) and actual vaccination rates (e.g., 25.43%) (10). Notably, intention and uptake are significantly higher among females, reflecting the vaccine's primary association with cervical cancer prevention and targeted health messaging (10).

**Influenza vaccine (IFV):** Influenza vaccine (IFV) is widely recognized as the most effective strategy against influenza, and vaccination among healthcare workers (HCWs) is strongly advocated (11). However, vaccine hesitancy persists even within this group, a situation potentially influenced by the complex legacy of the COVID-19 pandemic on vaccine attitudes and behaviors (12). Surveys in China show fluctuating but often suboptimal rates among medical staff (e.g., 22.6% in 2021–2022, rising to 56.7% in 2022–2023) (13), indicating room for improvement despite advocacy.

**Herpes Zoster vaccine (HZV):** This vaccine offers significant benefits in reducing HZ incidence, even in immunocompromised individuals, demonstrating good immunogenicity and cost-effectiveness (14). However, coverage rates in China remain markedly low and exhibit considerable disparity. Studies report rates as low as 13.26% among older adults in Beijing (with over half willing but unvaccinated) (15), and although increasing in Ningbo (e.g., cumulative coverage from 0.16% in 2020 to 2.97% in 2023), they still fall far short of rates seen in developed countries (16).

**Hepatitis B vaccine (HepBV):** A highly successful and safe intervention, HepBV is fundamental to preventing liver disease and blocking mother-to-child transmission (17). Indeed, it is one of the most direct methods for preventing liver cancer. China's integration of neonatal HepBV into the routine immunization schedule since 1992 represents a major public health success, dramatically reducing the under-five infection rate from 9.7% to below 1% (18).

Despite the proven efficacy of these vaccines and China's success with programs such as HepBV, the full potential of vaccination programs for HPV, influenza, HZV, and sustaining HepBV coverage is often constrained by persistent vaccine hesitancy within segments of the population (19). Therefore, a systematic analysis of vaccine acceptance and its determinants, specifically concerning these four key vaccines, among Chinese residents, is imperative. Such analysis is not only essential for refining targeted vaccination strategies from a scientific perspective but is also crucial for achieving the broader goals of the “Healthy China 2030” initiative. This study aims to address this need by assessing vaccine acceptance through the lens of actual vaccination behavior for HPV, IFV, HZV, and HepBV; identifying key influencing factors; and developing evidence-based strategies to enhance public uptake. These efforts will provide critical insights for strengthening China's vaccination programs and advancing public health outcomes.

## 2 Materials and methods

### 2.1 Study design and population

This cross-sectional study was conducted using data from the Psychology and Behavior Investigation of Chinese Residents (PBICR). PBICR is a large-sample, multicenter, replicated national study. The data were collected from 20 June 2023 to 31 August 2023 in China. The study population included 25 provinces, 5 autonomous regions, and 4 municipalities directly under the central government, totaling 150 cities. A total of 6 rural communities (villages) and 4 urban communities were sampled from each city, totaling 480 rural communities (villages) and 320 urban communities, for a total of 800 communities. Stratified random sampling was used at the provincial, community, or village level, and quota sampling was used at the community, village, or individual level. The questionnaire used in this study underwent 42 expert consultations and 3 pre-surveys to ensure the validity of the results. The study was approved by the Ethics Review Committee of Shandong Provincial Hospital (SWYX:NO. 2023-198) and is currently registered with the China Clinical Trial Registry (registration number: ChiCTR2300072573). All participants obtained written informed consent before participating in the survey.

The survey population included participants who met the following inclusion criteria: aged ≥18 years; with nationality of the People's Republic of China; permanent residents of China (annual time away from home ≤ 1 month); able to complete the online questionnaire on their own or with the help of an enumerator; and able to understand the meaning of the questionnaire as expressed in each entry. The exclusion criteria included the following: (1) people with delirium or mental abnormality; (2) people with cognitive dysfunction; (3) people who are participating in other similar research projects or people who have participated in previous years' surveys of PBICR; and (4) people who are unwilling to take part in this survey.

### 2.2 Research tools

#### 2.2.1 The eHEALS-SF scale

The eHEALS-SF scale was utilized to evaluate the e-health literacy level of the study participants. Developed by the PBICR project team based on the original eHEALS scale, this scale consists of five items (20). The adaptability of this scale has been validated in the Chinese population. These items cover aspects such as knowing where to find useful health resources online, being able to identify such resources, having the ability to use online health information for self-help, being capable of assessing the quality of online health resources, and having confidence in making informed health decisions using online information. Each item is scored on a 5-point Likert scale, with 1 indicating “strongly disagree”, 2 “disagree”, 3 “neutral”, 4 “agree”, and 5 “strongly agree”. In this study, the Cronbach's α coefficient for this scale was 0.883, indicating good internal consistency.

#### 2.2.2 Health literacy scale (HLS-SF4)

The Health literacy scale (HLS-SF4), a simplified version of the original Health Literacy Scale developed by the PBICR project team, was employed to measure the health literacy level of the participants (21), and the adaptability of this scale has been validated in the Chinese population. It contains four items. These items involve identifying treatment information for diagnosed conditions, evaluating the advantages and disadvantages of various treatment options, finding information on dealing with mental health issues such as stress or depression, and determining the types of vaccines one may need. The scale uses a 4-point Likert scale for scoring, where 1 represents “very easy”, 2 “easy”, 3 “difficult”, and 4 “very difficult”. With a Cronbach's α coefficient of 0.842 and a split-half reliability of 0.815, the scale demonstrates acceptable reliability for assessing health literacy in this study.

#### 2.2.3 Big five inventory-10 (BFI-10)

The Big five inventory-10 (BFI-10) is a concise five-factor personality assessment tool designed to rapidly evaluate the tendencies of study participants across five core personality dimensions: openness, conscientiousness, extraversion, agreeableness, and neuroticism (22). Each dimension is measured by two items (one positively worded and one negatively worded). Scoring is carried out on a 5-point Likert scale, ranging from 1 = “Completely Disagree” to 5 = “Completely Agree”. For negatively worded items, reverse scoring is applied. The Cronbach's α coefficients for each dimension of this scale range between 0.50 and 0.80, suggesting moderate-to-good internal consistency for measuring these personality traits.

#### 2.2.4 General risk tendency scale (GRiSP)

The General risk tendency scale (GRiSP), introduced by the PBICR project team, is a unidimensional scale that comprehensively assesses an individual's propensity to take external risks through multiple items, and the adaptability of this scale has been validated in the Chinese population. It consists of eight items, such as “Taking risks makes life more engaging”, “My friends perceive me as someone who takes risks”, and others. Each item is scored on a 5-point Likert scale, with 1 = “strongly disagree”, 2 = “somewhat disagree”, 3 = “neither agree nor disagree”, 4 = “somewhat agree”, and 5 = “strongly agree”. In this study, the Cronbach's α coefficient for this scale was 0.922, indicating excellent internal consistency for measuring an individual's risk-taking tendency.

#### 2.2.5 Media motivation scale

This scale, developed by the PBICR project team (23), is designed to evaluate the primary types of motivation among study participants when using social media or other digital platforms. The adaptability of this scale has been validated in the Chinese population. It includes five items related to information seeking, information sharing, self-status pursuit, social interaction, and relaxation and entertainment. Scoring is done on a 5-point Likert scale, from 1 = “strongly disagree” to 5 = “strongly agree”. In this study, the Cronbach's α coefficient for this scale was 0.893, showing good internal consistency in measuring media-related motivations.

### 2.3 Statistical analysis methods

After the questionnaires were collected, two individuals independently conducted logical consistency checks and data screening. The criteria for questionnaire exclusion were as follows: questionnaires with inconsistent logical responses; duplicate submissions; questionnaires containing incomplete information; and questionnaires in which all selected options were identical or followed a discernible pattern.

Data analysis was performed using SPSS 26.0 software. Continuous variables were assessed for normality using the Shapiro–Wilk test and visual inspection of histograms and Q-Q plots. Based on these assessments, continuous variables were presented as means ± standard deviations. Categorical variables were presented as frequencies or proportions. Univariate analysis was conducted using the chi-squared test, Freeman-Halton test, or Fisher's exact probability test, as appropriate. A logistic regression model was employed to analyze the influencing factors of vaccine acceptance among Chinese residents, with vaccination behavior (presence or absence) as the dependent variable and factors with *P* ≤ 0.01 in the univariate analysis as independent variables. During data cleaning, questionnaires with > 10% missing items were excluded, and for questionnaires with <10% missing data, continuous variables (e.g., scale scores) were interpolated using the means of the corresponding group (stratified by age and education), and categorical variables (e.g., occupations) were interpolated using the plurality. Comparison of the results before and after interpolation showed no significant differences (*p* > 0.05), confirming the robustness of the interpolation method. Prior to fitting the final logistic regression model, potential multicollinearity among the independent variables was assessed using the variance inflation factor (VIF). Variables with a VIF ≥ 5 were considered indicative of problematic multicollinearity and were evaluated for potential exclusion or combination. All statistical tests in this study were two-tailed, and a significance level of *P* < 0.05 was adopted.

## 3 Results

### 3.1 Basic information of survey respondents

This study included a total of 30,054 participants selected from the PBICR 2023 cross-sectional dataset. Following data cleaning procedures, the final analytic sample comprised 30,011 participants. Detailed demographic characteristics are summarized in Table 1.

**Table 1:** Characteristics of the survey respondents.

**Variables**	**Group**	**Amount**
Gender	Male	14,976
	Female	15,035
Age (years)	≤ 44	15,374
	45~59	8,900
	≥60	5,737
Ethnicity	Han ethnic group	27,294
	Minority group	2,717
Religious belief	Yes	2,272
	No	27,739
Educational states	Bachelor's degree or above	10,606
	Below undergraduate level	19,405
Occupational status	Students	4,681
	Employed	13,267
	Others	12,063
Suffering from illness	Yes	9,565
	No	20,446
Residence	Urban and Towns	20,715
	Rural	9,296
Marital status	Married	19,394
	Others	10,617
Monthly per capita income in family	≤ 5,000	18,210
(MPCI)	>5,000	11,801

### 3.2 Results of univariate analysis of vaccine acceptance among Chinese residents

#### 3.2.1 The overall acceptance of vaccines and influencing factors

Four vaccines (HPV, IFV, HZV, and hepatitis vaccines) were included in this research. Survey respondents who had received at least one kind of vaccine were regarded as having vaccination behavior and coded as 1, while others were coded as 0. The overall vaccination status was used as the dependent variable, and demographic characteristics and the scores scaled by different research tools were used as independent variables for univariate analysis. The results are summarized in Tables 2, 3. Among the 30,011 participants included in this study, 57.23% (*n* = 17,176) had received vaccinations, while 42.77% (*n* = 12,835) had not. The univariate analysis indicated that gender, age, educational attainment, occupational status, disease history, residential area, marital status, monthly per capita family income, eHEALS-SF scores, HLS-SF4 scores, agreeableness scores, conscientiousness scores, neuroticism scores, openness scores, GRiSP scores, and media motivation scale scores were all significantly associated with vaccination behavior (*P* < 0.05).

**Table 2:** Univariate analysis of demographic characteristics and vaccination status among Chinese residents.

**Variables**	**Group**	**Vaccination behavior**		***t/F***	***P***
		**Yes**	**No**		
Gender	Male	8,276 (27.58)	6,700 (22.33)	47.265	<0.001
	Female	8,900 (29.66)	6,135 (20.44)		
Age (years)	≤ 44	9,466 (31.54)	5,908 (19.69)	274.339	<0.001
	45–59	4,853 (16.17)	4,047 (13.49)		
	≥60	2,857 (9.52)	2,880 (9.60)		
Ethnicity	Han ethnic group	15,636 (52.10)	11,658 (38.85)	0.348	0.555
	Minority group	1,540 (5.13)	1,177 (3.92)		
Religious belief	Yes	1,256 (4.19)	1,016 (3.39)	3.735	0.053
	No	15,920 (53.05)	11,819 (39.38)		
Educational states	Bachelor's degree or above	7,077 (23.58)	3,529 (10.86)	603.436	<0.001
	Below undergraduate level	10,099 (33.65)	9,306 (31.01)		
Occupational status	Students	3,017 (10.05)	1,664 (5.54)	365.859	<0.001
	Employed	8,035 (26.77)	5,232 (17.43)		
	Others	6,124 (20.41)	5,939 (19.79)		
Suffering from illness	Yes	5,790 (19.29)	3,775 (12.58)	62.298	<0.001
	No	11,386 (37.94)	9,060 (30.19)		
Residence	Urban and towns	12,482 (41.59)	8,233 (27.43)	249.368	<0.001
	Rural	4,694 (15.64)	4,602 (15.33)		
Marital status	Married	10,637 (35.44)	8,757 (29.18)	127.178	<0.001
	Others	6,539 (21.79)	4,078 (13.59)		
MPCI	≤ 5,000	9,979 (33.25)	8,231 (27.43)	111.723	<0.001
	>5,000	7,197 (23.98)	4,604 (15.34)		

**Table 3:** Univariate analysis of different scores and vaccination status among Chinese residents.

**Variables**	**Vaccination behavior**		***t/F***	***P***
	**Yes**	**No**		
eHEALS-SF scores	17.81 ± 4.85	16.87 ± 5.18	−15.965	<0.001
HLS-SF4 scores	10.58 ± 2.66	10.01 ± 2.78	−17.942	<0.001
Extraversion scores	6.63 ± 1.37	6.65 ± 1.38	1.120	0.263
Agreeableness scores	6.34 ± 1.39	6.31 ± 1.45	−2.027	0.043
Conscientiousness scores	6.49 ± 1.38	6.42 ± 1.40	−4.271	<0.001
Neuroticism scores	6.55 ± 1.42	6.51 ± 1.45	−2.420	0.016
Openness scores	6.54 ± 1.42	6.60 ± 1.45	3.540	<0.001
GRiSP scores	23.82 ± 7.00	23.51 ± 7.21	−3.654	<0.001
The media motivation scale scores	18.06 ± 4.39	16.76 ± 4.85	−23.980	<0.001

#### 3.2.2 The acceptance of specific vaccines and influencing factors

Among the 30,011 Chinese residents surveyed in this study, 4,555 (15.18%) received the HPV vaccine, 12,103 (40.33%) received the IFV, 2,450 (8.16%) received the HZV, and 9,172 (30.56%) received the hepatitis vaccine. For further analysis of the specific vaccines used in the Chinese population, each vaccine was taken as the dependent variable (0 = no, 1 = yes), and univariate analysis was conducted as described before. The factors are summarized in Table 3. The results revealed that gender, age, educational attainment, occupational status, disease history, residential area, monthly per capita family income, eHEALS-SF scores, HLS-SF4 scores, GRiSP scores, and media motivation scale scores significantly influenced each vaccine's acceptance among Chinese residents, which is consistent with the overall analysis. However, other factors showed different effects on specific vaccines. Ethnicity was only associated with HZV and hepatitis vaccines. Religious belief was a significant factor for IFV, HZV, and hepatitis vaccines but not for HPV vaccination. Regarding personality influence, the five core personality dimensions varied across different vaccines. Extraversion, openness, and conscientiousness scores were significantly related only to HPV vaccination, while agreeableness and neuroticism scores were significantly related only to the hepatitis vaccine (Table 4).

**Table 4:** Univariate analysis of influencing factors among specific vaccines.

**Variables**	**HPV**		**IFV**		**HZV**		**Hepatitis vaccine**	
	***t/F***	***P***	***t/F***	***P***	***t/F***	***P***	***t/F***	***P***
Gender	890.661	<0.001	11.952	0.001	32.356	<0.001	9.582	0.002
Age (Years)	614.924	<0.001	64.975	<0.001	9.275	0.010	91.328	<0.001
Ethnicity	1.538	0.205	1.205	0.263	7.268	0.007	12.166	<0.001
Religious belief	0.593	0.432	11.359	0.001	16.534	<0.001	8.870	0.003
Educational states	702.862	<0.001	149.828	<0.001	20.101	<0.001	230.416	<0.001
Occupational status	471.421	<0.001	151.816	<0.001	11.785	<0.001	194.164	<0.001
Suffering from illness	51.200	<0.001	54.028	<0.001	72.841	<0.001	73.231	<0.001
Residence	188.326	<0.001	35.595	<0.001	13.436	<0.001	231.582	<0.001
Marital status	171.381	<0.001	89.224	<0.001	30.748	<0.001	0.015	0.891
MPCI	232.913	<0.001	19.081	<0.001	53.900	<0.001	128.484	<0.001
eHEALS-SF scores	19.048	<0.001	11.983	<0.001	7.138	<0.001	14.681	<0.001
HLS-SF4 scores	18.040	<0.001	12.952	<0.001	8.799	<0.001	14.217	<0.001
Extraversion scores	−4.241	<0.001	1.503	0.133	0.195	0.846	1.590	0.112
Agreeableness scores	1.402	0.161	0.147	0.883	−1.115	0.265	4.144	<0.001
Conscientiousness scores	6.310	<0.001	2.270	0.023	0.332	0.740	3.416	0.001
Neuroticism scores	2.312	0.021	1.194	0.232	−0.199	0.843	4.520	<0.001
Openness score	−4.606	<0.001	−2.304	0.021	−1.788	0.074	−0.345	0.730
GRiSP scores	6.692	<0.001	2.683	0.007	8.894	<0.001	7.122	<0.001
The media motivation scale scores	19.831	<0.001	15.458	<0.001	5.090	<0.001	20.709	<0.001

### 3.3 Logistic regression analysis of vaccine acceptance among Chinese residents

Taking whether Chinese residents had engaged in vaccination behavior as the dependent variable (0 = no, 1 = yes), factors with *P* < 0.05 in the univariate analysis were included as independent variables in the logistic regression model. Age was categorized as a dummy variable, and the remaining independent variables were assigned numerical values. The Electronic Health Literacy Scale, the Health Literacy Scale, the Big Five Personality Scale, the General Risk Propensity Scale, and the Media Motivation Scale were incorporated using their original scores. The results of the logistic regression analysis revealed that gender, age, educational level, employment status, disease history, place of residence, per capita monthly household income, health literacy, openness personality trait, risk acceptance tendency, and media motivation significantly influenced vaccine acceptance among Chinese residents, with statistically significant differences (*P* < 0.05). These results are summarized in Table 5. Further logistic analysis was also conducted for each vaccine separately. The results showed no differences between each vaccine, except for the age factor. The age (≥60) group showed no significant association with HPV (*P* = 0.262), while the age (45–59) group showed no significant association with HPV (P=0.206). Moreover, when we include the administration other three vaccines as independent variables in the logistic regression model, significant relations can be found between each other. Residents who have received IFV are more likely to receive HPV (OR=1.414, 95%CI: 1.344–1.546), HZV (OR=3.253, 95%CI: 2.938–3.602), and hepatitis vaccines (OR=3.144, 95%CI: 2.981–3.316) compared to those who have not received IFV. The same trends can be found in each vaccine.

**Table 5:** Logistic regression analysis of vaccine acceptance among Chinese residents.

**Projects**	**β**	**SE**	**Wald χ^2^**	***P***	**OR (95% CI)**
Constant term	−0.549	0.100	30.377	0.000	
Gender	−0.182	0.024	56.403	0.000	0.834 (0.795–0.874)
Age (≤ 44)			23.487	0.000	
Age (45–59)	0.169	0.043	15.735	0.000	1.184 (1.089–1.287)
Age (≥60)	0.032	0.038	0.722	0.395	1.033 (0.959–1.113)
Educational states	−0.397	0.030	177.519	0.000	0.672 (0.634–0.713)
Occupational status	−0.101	0.022	21.521	0.000	0.904 (0.866–0.943)
Suffering from illness	0.608	0.030	414.499	0.000	1.837 (1.733–1.948)
Residence	−0.175	0.027	41.630	0.000	0.840 (0.796–0.885)
Monthly per capita family income	0.073	0.026	8.008	0.005	1.076 (1.023–1.131)
HLS-SF4 scores	0.045	0.005	89.343	0.000	1.046 (1.036–1.056)
Openness scores	−0.021	0.009	6.085	0.014	0.979 (0.962–0.996)
GRiSP scores	−0.006	0.002	9.524	0.002	0.994 (0.991–0.998)
The media motivation scale scores	0.050	0.003	273.796	0.000	1.051 (1.045–1.058)

## 4 Discussion

### 4.1 The role of personal characteristics

#### 4.1.1 Age and health status

The findings of this study indicate that individuals aged 45–59 exhibit the highest vaccination willingness, which is contrary to the results reported by Sweta (24). From both physiological and social-role perspectives, this age group is at the onset of a high-disease-incidence period. As they age, their physical functions gradually decline, making them more sensitive to potential health threats and significantly increasing their focus on personal health. In addition, within the family structure, individuals in this age group often shoulder significant responsibilities. They are not only tasked with caring for the aged parents but also with ensuring the wellbeing of their children. Therefore, to safeguard the overall health of the family, they actively seek effective methods for preventing infectious diseases, and vaccination has emerged as a crucial disease-prevention option. However, individuals aged 60 and above showed no significant relation with vaccination willingness. As described before, the acceptance of vaccination among the aged remains relatively stable (25), and their relatively weaker physical functions make them more worried about vaccines' side effects (26, 27).

Furthermore, individuals with a history of disease have a significantly higher vaccination willingness, which is in line with the results of previous studies by Chen (28). Previous health challenges have enabled this group to have a deeper understanding of disease risks. Through their personal experiences of illness, they have not only endured the discomfort and inconvenience caused by diseases but also recognized the profound impact of diseases on quality of life and physical wellbeing. This firsthand experience has significantly strengthened their preventive awareness, which is then translated into proactive vaccination behaviors. Compared with those without a prior illness, individuals with a history of disease have more confidence in medical interventions and are more inclined to adopt preventive measures to reduce the risk of disease recurrence. For example, individuals who have previously contracted influenza are more likely to seek vaccination when they learn that the flu vaccine can effectively reduce the likelihood of reinfection.

#### 4.1.2 Educational attainment and health literacy

This study has identified individuals with high educational attainment as well as the high health literacy are more likely to accept vaccines, which is in line with the previous research (29–31). Those with high educational attainment and health literacy possess extensive health knowledge, enabling them to recognize the importance of vaccination in disease prevention and to have a scientifically based understanding of vaccine safety and efficacy. Vaccination involves complex aspects such as the mechanism of action, safety, and efficacy of vaccines. Those with lower educational and health literacy levels often find it difficult to understand these scientific principles, making it challenging for them to professionally evaluate the benefits and risks of vaccination. For example, individuals with lower educational levels may rely heavily on interpersonal communication, such as discussions with family and friends, which often lacks professionalism and accuracy, thus contributing to information bias. In contrast, individuals with higher educational attainment are more likely to actively seek information from reliable sources, including formal media, professional literature, and academic research. This enables them to develop a comprehensive and scientifically informed understanding of vaccines, increasing their willingness to vaccinate.

#### 4.1.3 Psychological and behavioral determinants

Regarding personality traits, individuals with a high openness score show a lower willingness to be vaccinated, which contradicts the findings of previous studies (32). It is generally assumed that open individuals are more accepting of novel experiences. However, in the context of vaccination, the situation is more complex (33, 34). The concept of openness has dual characteristics. On the one hand, individuals with an open personality may be more receptive to new technologies and concepts, including those related to vaccines. On the other hand, their strong critical-thinking skills may lead them to question authority, potentially resulting in skepticism toward vaccine-related information. During the dissemination of vaccine-related content, unverified negative information may be over-interpreted by open individuals, causing them to align with certain anti-vaccine perspectives and develop resistance toward vaccination. For example, individuals with a high level of openness may closely examine the vaccine's research and development process and safety data. Without fully understanding the underlying scientific rationale, they may ultimately refuse vaccination.

Another determinant on the willingness to be vaccinated is the risk orientation. It aligns with the “Protection Motivation Theory” (35). Risk-averse individuals tend to be more sensitive to potential risks and view vaccination as an effective strategy for reducing the likelihood of contracting infectious diseases. In the face of infectious disease threats, such individuals are more likely to choose vaccination as a preventive measure to safeguard their own health and that of their families. Conversely, individuals with a higher risk appetite may underestimate the severity and probability of disease contraction, showing insufficient awareness of the necessity of vaccination. They are more likely to accept potential risks, believing that they may remain uninfected even without vaccination. For example, some young adults, due to their perception of robust physical health and low susceptibility to diseases, may choose not to vaccinate based on their risk-oriented preferences.

#### 4.1.4 Economic conditions and geographical determinants

The results of this study show that individuals with higher household incomes have a higher vaccination rate. From an economic perspective, a higher household income means greater access to financial resources that can be allocated to health-security investments. Such households can not only afford the direct costs associated with vaccinations but also have the means to access vaccination-related information and transportation to vaccination sites (36). Rural areas face unique structural barriers possibly due to the fact that there are fewer vaccination sites in rural areas, which increases travel time and costs. Vaccine storage capacity in rural health facilities is limited, leading to frequent stockouts of non-routine vaccines (e.g., shingles vaccine), which directly reduces accessibility. In addition, rural vaccination subsidies (e.g., transportation subsidies) are not well developed, making the financial barriers faced by low-income groups even more pronounced.

In terms of geographic distribution, urban areas are characterized by a dense network of medical facilities and a wide availability of vaccination sites. Urban residents benefit from convenient appointment systems, including online platforms and community notifications, which enhance accessibility. Consistent with Korkmaz's research (37), this study found that the vaccination rate among urban populations is higher. This is because the well-developed transportation infrastructure in urban areas reduces both the time and economic costs for residents to travel to vaccination sites. Conversely, rural areas face distinct challenges. Remote vaccination sites may require rural residents to incur significant time and transportation expenses, potentially dampening their motivation to seek vaccinations.

### 4.2 The role of HBM and TPB in explaining vaccination behavior

The findings of this study align with the core propositions of the Health Belief Model (HBM) and Theory of Planned Behavior (TPB), providing empirical support for their utility in explaining vaccination behavior among Chinese residents.

#### 4.2.1 From the perspective of HBM

The significant association between disease experience and higher vaccination rates (OR = 1.837, 95%CI: 1.733–1.948) directly reflects HBM's assertion that personal experience enhances perceived susceptibility to disease, motivating preventive behavior. Individuals with a history of illness are more aware of health risks, which strengthens their willingness to vaccinate. Urban residents (OR = 0.840, 95%CI: 0.796–0.885) and those with high income (OR = 1.076, 95%CI: 1.023–1.131) exhibit higher vaccination rates as they face fewer logistical and economic barriers. This supports HBM's emphasis on balancing perceived benefits (disease prevention) against barriers (cost, accessibility). High media motivation (OR = 1.051, 95%CI: 1.045–1.058) acts as an external cue, prompting individuals to seek information and convert intentions into behavior, consistent with HBM's framework.

#### 4.2.2 From the perspective of TPB

High health literacy (OR = 1.046, 95%CI: 1.036–1.056) is associated with positive attitudes toward vaccination as individuals with better health cognition can better understand the scientific basis of vaccines and form favorable evaluations. Students, as a group with high vaccination rates, are likely influenced by institutional norms (e.g., school health promotion), reflecting TPB's focus on social pressure shaping behavior. Rural residents show lower vaccination rates due to limited access to services, which aligns with TPB's assertion that low perceived control inhibits behavior.

Together, HBM and TPB provide a comprehensive theoretical lens to interpret the multi-factorial nature of vaccination behavior. HBM explains how risk perception and practical barriers drive behavior, while TPB complements this by highlighting the role of psychological traits and social influences, collectively enhancing the explanatory power of our findings.

### 4.3 The role of media and information environment

Strong media motivation indicates that individuals actively seek information through various channels, including official policies and guidelines, professional scientific articles, and authoritative public health resources. The acquisition of such information helps to bridge cognitive gaps regarding vaccines and facilitates more rational decision-making regarding vaccination. However, the media plays a pivotal role in shaping vaccine hesitancy by amplifying misinformation, reinforcing echo chambers, and influencing public trust in credible VS. non-credible sources. Misinformation on social media (e.g., false narratives about vaccine side effects) can distort an individual's perception of risk, thereby significantly reducing willingness to vaccinate. Misinformation creates an “information asymmetry” that makes groups with lower health literacy more likely to accept false narratives (e.g., beliefs that vaccines cause chronic diseases). This amplifies perceived barriers in the HBM, which inhibits vaccination behavior. Cross-tabulation analyses of media attention and vaccination rates showed that individuals with high media attention but low health literacy had a 12.3% lower vaccination rate than those with high health literacy, suggesting that misinformation undermines the positive impact of media engagement. Social media platforms have emerged as dominant channels for health information, yet they also serve as vectors for anti-vaccine narratives and conspiracy theories. Driven by the proliferation of false claims about vaccine safety, higher hesitancy rates on vaccination, which related with social media, were found in different studies (38, 39).

In the current information-driven era, it is essential to fully utilize the media-engagement capabilities of individuals with a high motivation for media interaction. Given the extensive popularity of social media and short video platforms, these channels have emerged as a crucial means of information dissemination. To enhance public awareness of vaccines, it is necessary to develop scientifically accurate and accessible vaccine-related content. Concise expert interview clips can also be produced, where experts explain key aspects of vaccines, such as their efficacy, safety, and importance in disease prevention. By encouraging highly engaged individuals on social media to share and spread this content, a wider audience can gain access to vaccination-related knowledge. For groups with open personalities, who typically have higher demands for information quality, evidence-based medical data should be provided. This can be achieved by organizing experts to write professional science communication articles. These articles should detail aspects such as the vaccine development process, clinical trial results, and safety evaluations. By presenting robust scientific evidence, these articles can alleviate irrational concerns among the public and increase their acceptance of vaccination. This approach not only provides accurate information but also helps to build public trust in vaccines, which is essential for promoting higher vaccination rates.

### 4.4 The differences in influencing factors among specific vaccines and their interactive effects

#### 4.4.1 The differences in influencing factors among specific vaccines

While many research studies have identified various factors influencing people's vaccination behavior, limited prior work focused on the comparison between different vaccines, and no work has examined the interaction between different vaccines in the Chinese population. Our analysis, based on a survey of Chinese residents, finds that, although the four vaccines are all recommended by the government for protection against viruses, the prevalence and acceptance rate are discordant in the Chinese population. Furthermore, each vaccine has specific influencing factors. First, religious beliefs and ethnicity frequently contribute to the vaccination hesitancy (40, 41), but in our research, they only influence the HZV and hepatitis vaccine. Second, psychological and behavioral determinants significantly influence vaccine hesitancy, shaping how individuals perceive and respond to vaccination campaigns. Personality traits, such as low openness to experience and high paranoia, also correlate with hesitancy, highlighting how individual differences influence vaccination attitudes also influences hesitancy (42). However, as shown in our research, the same personality trait can play differing roles across different vaccines. This may be influenced by social and cultural factors, which warrant further investigation in future studies. Analyzing the factors affecting the rollout of each vaccine may help improve uptake and inform strategies and policies for future vaccine programs.

#### 4.4.2 Relationship between different vaccine experiences

Based on the Behavioral Spillover Theory, one specific vaccination history may lead to positive attitudes toward other vaccinations among the population. For example, people who have an influenza vaccination history may have higher motivation for pneumococcal vaccination (43). However, previous studies often focus on the vaccines acting on the same system, such as IFV and pneumococcal vaccines, IFV, and the COVID-19 vaccine (44). It seems insufficient for the verification of the relationship between different vaccinations and the spillover effects of vaccination history. In this study, influenza vaccination history may influence the other three vaccination behaviors. This result is consistent with previous studies, which found that IFV increased the likelihood of receiving pneumococcal vaccination. It may be partially because individuals who have received the IFV may exhibit greater health consciousness and possess a higher level of vaccine literacy. This, in turn, could lead to an increased likelihood of obtaining other vaccines as a means of self-protection. Furthermore, the fact that all the vaccines present significant interaction with each other provides strong proof of the previous theory.

### 4.5 Policy and practical implications

In light of the above-mentioned research findings, which identified significantly lower COVID-19 vaccine acceptance rates among individuals with low educational attainment, those residing in rural areas, and those with lower economic incomes, it is imperative to implement precision intervention strategies. For these key vulnerable groups, it is crucial to address the numerous challenges they encounter during the vaccination process, including limited access to information and logistical obstacles to getting vaccinated.

To mitigate these issues, several targeted measures can be adopted. First, community outreach efforts should be strengthened. Trained professionals (e.g., public health workers and trusted community leaders) can be dispatched to conduct vaccination awareness workshops within communities, potentially held at regular intervals in accessible community centers. During these workshops, the significance and safety of vaccinations should be explained in clear and easy-to-understand language. This approach helps to enhance residents' understanding of vaccines and their importance in disease prevention. Second, dialect-based science communication can be effectively utilized. By using local dialects to convey vaccination-related information through multiple channels such as village loudspeaker announcements, locally produced short videos distributed via popular social media apps (e.g., WeChat), and illustrated pamphlets, rural populations and individuals with lower educational levels can better comprehend and accept the content. This method bridges the communication gap and ensures that the information reaches the intended audience more effectively. Third, door-to-door services can be provided. This is particularly beneficial for residents with mobility limitations or those living in remote areas. Mobile vaccination teams, potentially coordinated through township health centers and utilizing pre-registration systems, can visit villages/households on scheduled days. Door-to-door vaccination services eliminate the need for these individuals to travel long distances to vaccination sites, thereby reducing logistical barriers and increasing vaccination accessibility.

Furthermore, our findings underscore the importance of tailored approaches for the aged population (aged ≥60 years). Integrating vaccination into comprehensive health management frameworks, such as family doctor contract services, is of great significance. Specifically, family doctors can proactively incorporate vaccination status review and personalized recommendation discussions into routine chronic disease management visits or annual health check-ups. Based on these consultations, they can provide personalized vaccination recommendations tailored to each the aged person's specific health conditions. This personalized approach not only promotes higher vaccination rates among the aged but also contributes to their overall health management and wellbeing.

## 5 Conclusion

In summary, the acceptance of vaccines among Chinese residents is comprehensively influenced by demographic characteristics, health cognition, personality traits, and socioeconomic factors. Among these, disease experience, health literacy, and mediator motivation serve as core factors that promote vaccination. These elements can enhance residents' awareness and acceptance of vaccines while facilitating vaccination behaviors. Therefore, when formulating vaccination policies, it is essential to adopt multi-dimensional intervention measures tailored to the characteristics of different groups. By improving the accessibility of health information, more individuals can obtain accurate vaccination guidance. Enhancing risk perception encourages residents to prioritize disease prevention, while optimizing vaccination services and reducing barriers can increase vaccination coverage rates and protect public health. However, this study employs a cross-sectional design. While this methodology enables the collection of substantial data at a specific time point and facilitates the analysis of relationships between various factors, it cannot establish causality. Future research could integrate longitudinal data and qualitative research methods to further investigate the dynamic interplay between personality traits and vaccine decision-making, thereby providing a more robust and comprehensive foundation for the development of vaccination policies.

## Data Availability

The raw data supporting the conclusions of this article will be made available by the authors, without undue reservation.
